# Near‐Infrared‐Responded High Sensitivity Nanoprobe for Steady and Visualized Detection of Albumin in Hepatic Organoids and Mouse Liver

**DOI:** 10.1002/advs.202202505

**Published:** 2022-07-19

**Authors:** Guofeng Liu, Jinsong Wei, Xiaoyu Li, Meng Tian, Zhenxing Wang, Congcong Shen, Wan Sun, Chonghui Li, Xuewen Li, Enguang Lv, Shizheng Tian, Jihua Wang, Shicai Xu, Bing Zhao

**Affiliations:** ^1^ Shandong Key Laboratory of Biophysics Institute of Biophysics College of Physics and Electronic Information Dezhou University Dezhou 253023 China; ^2^ State Key Laboratory of Genetic Engineering School of Life Sciences Zhongshan Hospital Fudan University Shanghai 200438 China; ^3^ Greater Bay Area Institute of Precision Medicine (Guangzhou) Fudan University Nansha District Guangzhou 511458 China

**Keywords:** hepatic organoids, living cell labeling, mouse liver, near‐infrared‐responded high sensitivity nanoprobe, protein quantification and imaging

## Abstract

Exploring the advanced techniques for protein detection facilitates cell fate investigation. However, it remains challenging to quantify and visualize the protein with one single probe. Here, a luminescent approach to detect hepatic cell fate marker albumin in vitro and living cell labeling with upconversion nanoparticles (UCNPs), which are conjugated with antibody (Ab) and rose bengal hexanoic acid (RBHA) is reported. To guarantee the detection quality and accuracy, an “OFF–ON” strategy is adopted: in the presence of albumin, the luminescence of nanoparticles remains suppressed owing to energy transfer to the quencher. Upon albumin binding to the antibody, the luminescence is recovered under near‐infrared light. In various bio‐samples, the UCNPs‐Ab‐RBHA (UCAR) nanoprobe can sense albumin with a broad detection range (5–315 ng mL^−1^). When applied to liver ductal organoid culture medium, the UCAR can monitor hepatocyte differentiation in real time by sensing the secreted albumin. Further, UCAR enables live imaging of cellular albumin in cells, organoids, and tissues. In a CCl_4_‐induced liver injury model, UCAR detects reduced albumin in liver tissue and serum. Thus, a biocompatible nanoprobe for both quantification and imaging of protein in complex biological environment with superior stability and high sensitivity is provided.

## Graphical abstract

Förster resonance energy transfer‐based UCNPs‐Ab‐RBHA (upconversion nanoparticles‐antibody‐rose bengal hexanoic acid, UCAR) nanoprobe adopts an “OFF–ON” strategy to detect target protein. Under near‐infrared light, UCAR nanoprobe enables protein quantification in vitro and target cell labeling in living cells, organoids, and mouse liver with superior stability and high sensitivity, showing great potential in protein/cell tracking in vivo and clinical diagnosis.

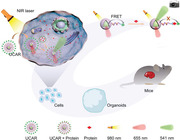

## 1 Introduction

As the downstream of genetic information flow in central dogma, functional proteins participate in all aspects of biological processes.^[^ ^1^ ^]^ Apart from the intrinsic characteristics, such as amino acid sequences and structures, the quantity and localization of proteins also determine the biochemical reactions, cell fates, and tissue structures. Thus, proteins have emerged as powerful biomarkers for indicating cell statuses related to developmental regulation, homeostasis maintaining, and pathological progression.^[^ ^2^ ^]^ Therefore, the protein detection techniques, such as enzyme‐linked immunosorbent assay (ELISA), immunofluorescence, and mass spectrum‐based detection methods, have achieved significant process in protein science. However, those techniques have several limitations, including tedious procedures, low repeatability, and limited living imaging applications.

Past few decades have witnessed the development of biosensors built with advanced nanomaterials. Nanoparticles provide superior characteristics, such as high reactivity, unique electrical and magnetic properties, and significant surface area to volume ratio.^[^ ^3^ ^]^ Compared with organic dyes and semiconductor quantum dots, rare earth‐doped upconversion nanoparticles (UCNPs) show superior chemical and optical properties, including large antistokes shift, narrow bandwidth emission, ultralow background, and high resistance to photobleaching.^[^ ^4^ ^]^ UCNPs also exhibit good biocompatibility and biosafety. Most UCNPs can enter cells via endocytosis, and the entering efficiency depends on the size, surface modification, and incubation time of nanoparticles.^[^ ^5^ ^]^ For instance, positively charged UCNPs, such as polyethyleneimine (PEI)‐UCNPs boosted the cellular uptake.^[^ ^6^ ^]^ Hyeon et al. reported that NaGdF_4_:Yb, Er nanoparticles (20 nm) need 4 h to enter the cells and release upcoversion luminescence.^[^ ^7^ ^]^ As the proof‐of‐concept for diagnosis and biomedical therapy, the UCNPs were administrated to animals. Through intravenous injection, the UCNPs were mainly distributed in liver and spleen.^[^ ^5^ ^]^ Live imaging revealed that intravenous injected UCNPs accumulated in liver at 60 min after injection, and peaked at 4 h.^[^ ^8^ ^]^ Moreover, histological analysis also proved no noticeable damage in major organs, including heart, liver, spleen, lung, and kidney,^[^ ^9^ ^]^ proving the low toxicity of UCNPs.

Since UCNPs could not detect the substrates, special recognition groups need to be attached onto the surface of UCNPs. Different strategies that utilized affinitive ligand, adaptor or peptide as recognition units have been reported.^[^ ^10^ ^]^ For biomarker detection, immuno‐affinitive UCNPs might be the best solution. He et al. designed a bispecific antibody‐UCNPs nanoprobe for the detection and imaging of cancer biomarker‐EphA2,^[^ ^11^ ^]^ but the background signal was relatively high and the preparation of bispecific antibody paid extra design and purification. Chen et al. adopted a sandwich structured platform by replacing the Horseradish Peroxidase (HRP) with UCNPs as a detection signal in Immunohistochemistry (IHC), which improved the detection limit, but still not suitable for real time monitoring. Despite tremendous efforts have been made in near‐infrared (NIR) fluorescence imaging,^[^ ^12^ ^]^ it is still challenging to quantify the biomarkers in vitro and label them in living cells with one single nanoprobe.

The albumin, mainly synthesized by hepatocytes in the liver and secreted into the blood system for circulation, accounts for about 50% of the total plasma protein and maintains body nutrition and osmotic pressure.^[^ ^13^ ^]^ Apart from its oncotic function, albumin is able to bind diverse molecules, including fatty acids, metabolites, drugs, and proteins, acting as toxin scavenger and chaperonins.^[^ ^14^ ^]^ Moreover, albumin is also the key indicator of many diseases, including cancer, rheumatoid arthritis, and liver cirrhosis.^[^ ^15^ ^]^ Most importantly, albumin is the classic biomarker for hepatic cell fate, and its expression level reflects the differentiation status of hepatocytes from its precursors or other lineages.^[^ ^16^ ^]^ Thus, quantification and imaging of albumin in biological context would be of great physiological and pathological significance.

**Scheme 1:**
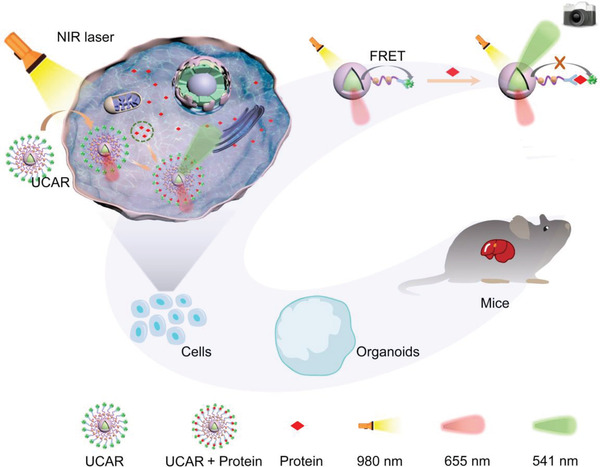
Schematic illustration of UCAR's mechanism for protein detection and its application in cells, organoids, and animals.

To detect albumin, we designed a nanoprobe called UCAR, which was composed of UCNPs, antibody (Ab), and rose bengal hexanoic acid (RBHA). RBHA was commonly used as a photosensitizer with many advantages, such as low toxicity, favorable biocompatibility, good stability, and visible light excitation wavelength.^[^ ^17^ ^]^ Due to the partial overlap of UCNP's emission spectrum and RBHA's absorption spectrum, the emission light of UCNPs could be quenched by RBHA in close distance based on Förster resonance energy transfer (FRET). Therefore, the upconversion luminescence would be restored when the nanoprobe binds albumin, which alters the distance between energy donor and quencher. The UCAR nanoprobe enables albumin detection in different liquid environment by using luminescence intensity and luminescence intensity ratio (LIR) analysis. Furthermore, the imaging and albumin detection also are achieved in HepG2 cells, hepatocyte organoids, and mouse liver under NIR laser irradiation (**Scheme** **1**).

## 2 Results

### 2.1 Designing an NIR‐Responded Nanoprobe for Albumin Sensing

We considered an albumin luminescence probe should 1) be photostable to avoid interference from photobleaching, ideally for the turn‐on probe, 2) has high signal‐to‐noise ratio, 3) applicable for deep tissue imaging, 4) has low cytotoxicity. Obviously, UCNPs would be a promising parent nanoprobe.

**Figure 1:**
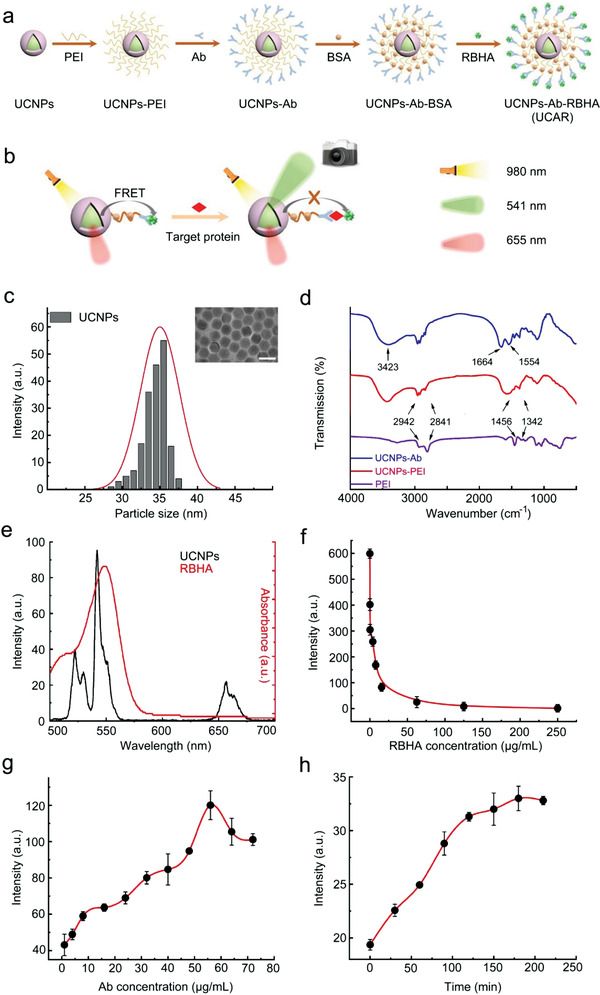
Synthesis and characterization of UCAR. a) The schematic diagram of UCAR synthesis process from NaYF_4_:Yb^3+^/Er^3+^@NaYF_4_ (UCNPs). b) The schematic diagram illustration of UCAR's mechanism for protein detection based on FRET. c) The size distribution and TEM image of UCNPs. The scale bar is 50 nm. d) The FTIR spectra of PEI, UCNPs‐PEI, and UCNPs‐Ab. e) The absorption spectrum of RBHA and the emission spectrum of UCNPs. f) The emission spectra of UCAR with different RBHA concentrations. g) The emission spectra of UCAR with different Ab concentrations. h) The change of luminescence intensities of UCAR after incubated with albumin for different incubation times. The statistical data were represented as mean ± S.D. (*n* = 3).

To improve the stability and reduce photobleaching,^[^ ^18^ ^]^ we first synthesized UCNPs as a core–shell structure with NaYF_4_:Yb^3+^/Er^3+^ as core and NaYF_4_ as inert shell (Figure S1, Supporting Information). Both core and core–shell‐structured UCNPs were monodispersed and have a uniform particle size (Figure S2 in the Supporting Information and **Figure** **1**c). The X‐ray diffraction (XRD) patterns of core and core–shell‐structured UCNPs were well matched the powder diffraction standard card (JCPDS#16‐0334), indicating the pure hexagonal phase of the UCNPs (Figure S3, Supporting Information). As expected, the luminescence intensity of UCNPs was greatly enhanced after coated with an inert shell (NaYF_4_) (Figure S4, Supporting Information).

The principle of energy transfer requires quencher to absorb the energy of donor, thus we measured the emission spectrum of UCNPs and the absorption spectrum of RBHA. Under 980 nm laser irradiation, the green (peak at 541 nm) luminescence of UCNPs clearly overlapped with RBHA's absorption spectrum (peak at 548 nm) (Figure 1e), suggesting FERT is logical.

The subsequent surface modification of UCNPs adopted step‐wised protocol illustrated in Figure 1a: The core–shell‐structured UCNPs were sequentially connected with PEI, antibody, bovine serum albumin (BSA), and RBHA. And the working logic would be: in the absence of target protein, the green emission light excited by 980 nm laser could be quenched by the proximal RBHA based on FRET, while the target protein is captured, the distance between UCNPs and RBHA would be altered, so the green emission light could be released and detected (Figure 1b). In technical details, the water‐soluble surface‐modifier PEI is loaded onto the surface of UCNPs by the ligand exchange method to form UCNPs‐PEI, and the antibody (Ab) and quencher (RBHA) are further connected by dehydration condensation between amino and carboxyl groups. The loading of PEI, Ab, and RBHA was confirmed by Fourier transform infrared absorption spectra (FTIR) and thermogravimetry (TG). The FTIR results showed stretching vibration of ‐CH_2_ (peaks at 2942, 2841 cm^−1^) and stretching vibration of ‐NH_2_ (peaks at 1456, 1342 cm^−1^), indicating the UCNPs have been effectively wrapped by PEI,^[^ ^19^ ^]^ and the peaks at 1664 and 1554 cm^−1^ are associated with the bending vibration of amide bonds,^[^ ^20^ ^]^ indicating that the Ab has been successfully loaded on UCNPs‐PEI (Figure 1d). In TG assay, the mass loss (8%) of UCNPs‐PEI (250 to 400 °C) in TG curve proved that a small amount of PEI was loaded on the surface of UCNPs, and the dramatic weight loss of UCNPs‐Ab (32.5%) and UCAR (54.5%) also indicated that Ab, BSA, and RBHA were carried on the surface of nanoparticles (Figure S6, Supporting Information). Further, the electrostatic adsorption also proved that the UCNP‐PEI and UCAR were positively charged, compared with RBHA, albumin, and antibody (Figure S7, Supporting Information). As expected, the luminescence intensity of UCNPs‐Ab could be ultimately quenched by 98% when the RBHA concentration was increased to 250 µg mL^−1^ (Figure 1f). By measuring the absorption curves of RBHA and UCAR (Figures S8 and S9, Supporting Information), the encapsulation rate and drug loading rate of RBHA were calculated as 95.5% and 4.3%, respectively. As the incubation time prolonged, the more RBHA would bind to UCNPS‐Ab, leading to the decrease of its luminescence intensity (Figure S10, Supporting Information).

In order to determine the optimal Ab concentration, we fixed albumin concentration at high concentration of 400 ng mL^−1^ and obtained the correlation between the luminescence intensity of the UCNPs‐Ab and Ab concentration. The luminescence intensity was increased with Ab concentration and climbed to the peak when the Ab concentration was reached to 56 µg mL^−1^ (Figure 1g). The optimal time for the combination of UCAR and albumin was also determined. At the fixed albumin concentration of 315 ng mL^−1^, the luminescence intensity of UCAR tends to be stable after incubated for 210 min (Figure 1h), demonstrating a full binding of albumin to UCAR. To quantify the albumin binding content, we incubated the constant UCAR (0.6 × 10^−3^ m, 100 µL) with increasing concentration of albumin, then measured the 541 nm intensity. Therefore, when the intensity reached at the top point, the maximum albumin binding quantity could be quantified (Figure S11, Supporting Information). According to the results, the saturated albumin concentration was about 350 ng mL^−1^, so the albumin binding content would be 0.93 g per mol UCAR nanoprobe.

To provide more details in physicochemical properties of nanoprobes, we performed serum stability test and cytotoxicity assay of UCAR. The dynamic light scattering data showed that diameters of UCAR nanoparticles barely changed in serum as long as 14 days (Figure S12, Supporting Information), suggesting good serum stability. Besides, Cell Counting Kit‐8 (CCK‐8) assay in HaCat and HepG2 cells showed cell survival rates were near 100% with the increasement of UCAR concentration (up to 500 µg mL^−1^), indicating the extremely low toxicity to cells (Figure S13, Supporting Information). The characterization and optimization of UCAR paved the way for its application in the following quantification and imaging experiments.

### 2.2 UCAR Nanoprobe Responds to Albumin in Linear Regression

Next, we tested the specificity of UCAR nanoprobe to albumin. Similar with the synthesis of UCAR, we manufactured the control nanoprobe consisted of UCNP‐IgG‐RBHA, which was abbreviated for UCIR. Then, the UCIR or UCAR was incubated with BSA, human IgG, albumin, albumin+BSA, and albumin+IgG, respectively. After incubation, the 541 nm luminescence intensity was recorded. The results showed that UCAR released significant higher luminescence intensity when incubated with albumin, compared with BSA and IgG, while the luminescence intensity of UCIR still remained background levels. When albumin is mixed with BSA or IgG, the fluorescence intensity of UCAR was not disturbed, compared with pure albumin (Figure S16, Supporting Information). To further prove the specificity of UCAR, we performed antibody neutralization experiments. By adding albumin antibody into UCAR‐albumin incubation system, we found that the released luminescence intensity dropped down to the control level with the increase of antibody concentration (Figure S17, Supporting Information), demonstrating the free antibody competed with UCAR to bind the albumin. The results further demonstrated good specificity and selectivity of this nanoprobe.

**Figure 2:**
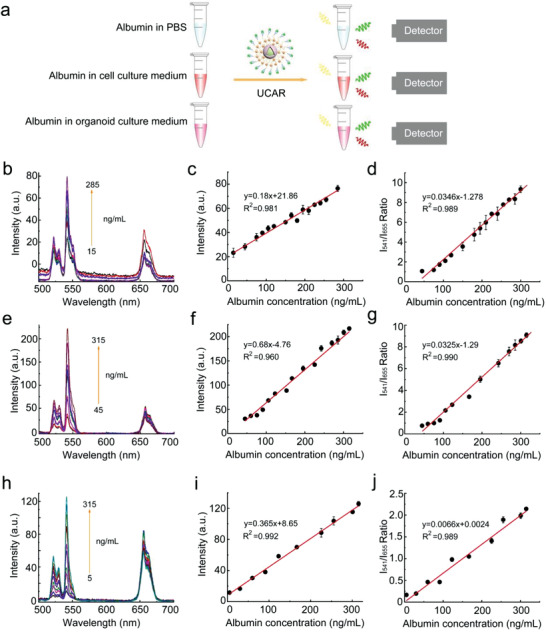
UCAR nanoprobe responds to albumin in linear regression. a) The illustration of UCAR‐mediated albumin detection in different solutions. b–d) The emission spectra, green luminescence intensity (*I*_541_), and LIR (*I*_541_/*I*_655_) of UCAR (0.6 × 10^−3^ m) in PBS solution after incubated with different albumin concentrations. e–g) The emission spectra, green luminescence intensity (*I*_541_), and LIR (*I*_541_/*I*_655_) of UCAR (0.6 × 10^−3^ m) in cell culture medium (DMEM containing 10% FBS) after incubated with different albumin concentrations. h–j) The emission spectra, green luminescence intensity (*I*_541_), and LIR (*I*_541_/*I*_655_) of UCAR (0.6 × 10^−3^ m) in organoid culture medium (advanced DMEM F12 supplemented with growth factors) after incubated with different albumin concentrations. The statistical data were represented as mean ± S.D. (*n* = 3).

We have observed the near‐linear correlation between the luminescence intensity of UCAR nanoprobe and antibody concentration (Figure 1g), so we reasoned that the luminescence intensity of UCAR and albumin concentration may also fit the linear regression, which is crucial for protein quantification. To address that, we dissolved the albumin standard sample in three different biological solutions (phosphate buffer solution (PBS), cell culture medium, and organoid culture medium), then added UCAR for luminescence intensity detection (**Figure** **2**a). Distinguished with PBS solution, the cell culture medium and organoid culture medium mimicked near‐physiological environment, which not only contain various nutrients, but also supplemented with serum or complex growth factors, such as R‐Spondin, epidermal growth factor (EGF), hepatocyte growth factor (HGF), fibroblast growth factor (FGF), and gastrin.^[^ ^21^ ^]^ The aim of different solution is to test the stability and specificity of UCAR nanoprobe.

Ideally, the UCAR probe responded to the albumin in different solutions with a wide detection range (5–315 ng mL^−1^). The emission spectra data showed that the 541 nm luminescence intensity dramatically increased with the albumin concentrations, while the 655 nm luminescence intensity was relatively stable (Figure 2b,e,h). By plotting 541 nm luminescence intensity values and albumin concentrations, we observed linear relationship between 541 nm luminescence and albumin concentration (Figure 2c,f,i). According to the equations of linearized regression, the detection limit of nanoprobe could be calculated as low as 0.053 ng mL^−1^.^[^ ^22^ ^]^ Since the RBHA solely quenched the green light (541 nm) of UCNPs (Figure 1d), the red light (655 nm) intensity could be measured as internal reference. Therefore, calculating the green/red LIR, *I*_541_/*I*_655_ might further exclude the dosage dependence of UCAR. Expectedly, the LIR also fit liner regression with albumin concentrations (Figure 2d,g,j). Collectively, the UCAR displayed excellent stability and reliability in complex biological samples for albumin quantification in vitro.

### 2.3 UCAR Enables Precise Quantification of Albumin Secreted by Liver Cells and Hepatic Organoids

**Figure 3:**
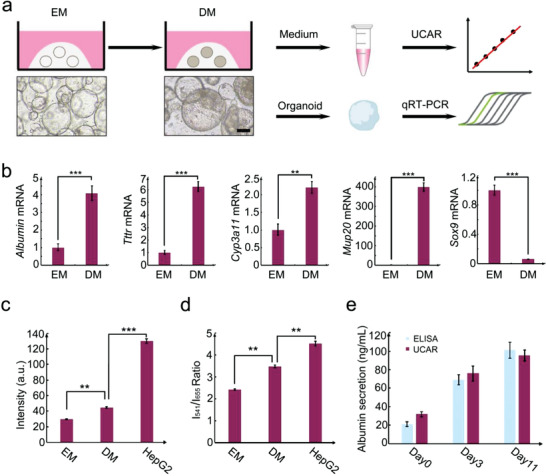
UCAR can detect the albumin secreted by living cells and hepatic organoids. a) The scheme of UCAR‐mediated albumin detection in liver organoid differentiation system. Scale bar = 100 µm. b) qRT‐PCR analysis of hepatocyte (*Albumin*, *Ttr*, *Cyp3a11*, and *Mup20*) and ductal (*Sox9*) cell markers in EM‐ and DM‐cultured liver organoids. *Histone H3* was used as an internal control. c,d) The albumin detection in vitro by using luminescence intensity of c) *I*_541_ and d) LIR of *I*_541_/*I*_655_ of UCAR (0.6 × 10^−3^ m) after incubated with different culture media of EM, DM, and HepG2. e) The albumin concentration of conditional medium collected at the day 0, day 3, and day 11 after the differentiation induction, is detected and compared by ELISA and UCAR. The statistical data represent mean ± S.D. (*n* = 3). **, *p* < 0.01. ***, *p* < 0.001.

Albumin is synthesized and secreted by hepatocytes, so we test whether UCAR could detect the albumin secreted by hepatic cells or organs. Liver bile duct‐derived progenitor cells can self‐assemble into long‐term expandable liver bipotent organoids in a 3D culture system with defined culture medium, which reserves the potential to differentiated to cholangiocytes and hepatocytes.^[^ ^23^ ^]^ When cultured in expansion medium (EM), the liver ductal organoids exhibit ductal cell fate, which secreted low‐level albumin. When cultured in differentiation medium (DM), the liver ductal organoid could differentiate toward hepatocyte, which secreted high‐level albumin. During the differentiation induction process (11 days), the secreted albumin gradually increases with the induction time. By monitoring the albumin expression and secretion level, the differentiation status of liver organoid could be determined.^[^ ^16^, ^21^ ^]^ Therefore, we followed the liver organoid differentiation protocol and collected the conditional medium and organoid in EM and DM stages, to test whether UCAR nanoprobe could monitor hepatocyte differentiation (**Figure** **3**a).

To validate the differentiation efficiency, quantitative real‐time polymerase chain reaction (qRT‐PCR) was conducted to determine the expression level of hepatocyte markers. As shown by Figure 3b, in DM organoids, the expression level of classical hepatic markers significantly increased, including *Albumin*, *Transthyretin (Ttr)*, cytochrome P450 family member *Cyp3a11*, *and* major urinary protein *Mup20*. In the meantime, the expression level of ductal marker SRY (sex determining region Y)‐box 9 (*Sox9*) dramatically dropped in DM, indicating the successful cholangiocyte‐to‐hepatocyte differentiation. We also quantified the secreted albumin using enzyme‐linked immunoassay (ELISA), the result also proved significant increase of albumin concentration in DM medium, compared with that of EM (Figure S14, Supporting Information).

Then the EM, DM, and HepG2 cells medium (positive control) were incubated with UCAR for 300 min, respectively. The results showed that the luminescence intensity (*I*_541_) and LIR (*I*_541_/*I*_655_) of culture media of DM and HepG2 cells are both higher than those of EM (Figure 3c,d), which were consistent with the previous qRT‐PCR (Figure 3b) and ELISA results (Figure S14, Supporting Information). To prove the accuracy of the UCAR‐mediated protein quantification, we compared the ELISA method and UCAR method, and the results showed that the albumin concentrations detected by UCAR method equal to ELISA method (Figure 3e), indicating that the UCAR method could substitute for ELISA with simplicity and accuracy.

### 2.4 UCAR Allows Albumin Imaging in Hepatic Cells and Organoids

**Figure 4:**
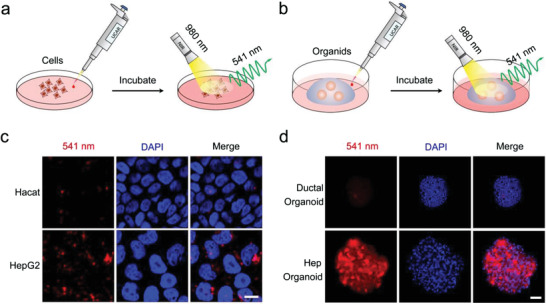
The bio‐imaging of albumin in HepG2 cells and hepatocyte organoids using UCAR. a,b) The schematic illustration of albumin imaging in a) cells and b) organoids using UCAR. The UCAR was directly added into the culture medium of living cells and organoids, after incubation, the cells or organoids were subjected to 980 nm irradiation and 541 nm signal was collected. c) HaCat cells and HepG2 cells were incubated with UCAR for 3 h, followed by fixation and DAPI staining, and imaged by two‐photon microscope under 980 nm excitation. The released 541 nm luminescence was shown by red color and 405 nm DAPI luminescence was shown by blue color. A representative result of three independent experiments was shown. The scale bar = 10 µm. d) Similarly, ductal organoids and hepatocyte organoids were incubated with UCAR for 3 h, followed by fixation and DAPI staining, and imaged by two‐photon microscope under 980 nm excitation. The released 541 nm luminescence was shown by red color, and 405 nm DAPI luminescence was shown by blue color. A representative result of three independent experiments was shown. The scale bar = 50 µm.

Living image of endogenous protein in cells and organs relies on the organic dyes or genetic‐modified fluorescent protein tags, which heavily limit the investigation of protein dynamics in living cells. Therefore, we test whether UCAR could image albumin in living cells and organoids.^[^ ^24^ ^]^ During cell or organoid culture, the UCAR was directly added into the culture medium and incubated for at least 3 h. Then the cells or organoids were fixed and stained with 4′,6‐diamidino‐2‐phenylindole (DAPI), followed by imaging (excitation: 980 nm, emission: 541 nm) (**Figure** **4**a,b). Compared with the mock group (adding PBS), the UCAR added group shows robust 541 nm luminescence in cell cytoplasm (Figure S15, Supporting Information). To further exclude the background signals and demonstrate the specificity, we selected HaCat (keratinocytes cell line) and ductal organoid as negative controls. The results showed that, under 980 nm irradiation, the HepG2 group exhibits strong 541 nm signal near the cell nucleus, indicating the cytoplasm location of albumin. While in HaCat group, only sporadic signals were observed (Figure 4c). Similar to the cell results, the hepatocyte organoids exhibit robust luminescence at 541 nm, while the ductal organoid shows near‐zero luminescence (Figure 4d). Those results indicated that the UCAR nanoprobe could be swallowed by the cells, and could bind to the albumin protein in cytoplasm, thus showing good biocompatibility for the living animal applications.

### 2.5 UCAR Reveals Dynamic Changes of Albumin in Mouse Liver

**Figure 5:**
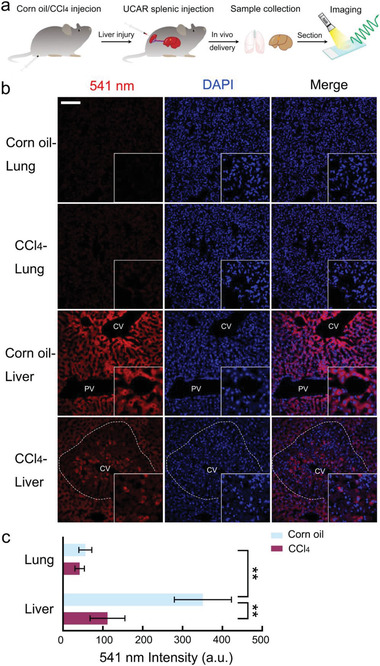
UCAR reveals dynamic changes of albumin in injured liver. a) The workflow of UCAR‐mediated albumin imaging in the liver injury model. The mice were treated with CCl_4_/corn oil to induce liver injury. After 24 h, mice were received 200 µL UCAR (2.5 mg mL^−1^) by splenic injection. After 4 h, mice were sacrificed for liver and lung sample collection and confocal imaging. b) The lung and liver samples collected from CCl_4_/corn oil‐treated mice were subjected to 980 nm excitation using two‐photon microscope. The UCNPs released 541 nm luminescence was shown by red color, and 405 nm DAPI fluorescence was shown by blue color. Magnification, x2. A representative result of three independent experiments is shown. Scale bar = 100 µm. The dash lines in CCl_4_‐liver image indicate zone 3 showing significant signal reduction and immune cells penetration. c) The quantification and statistical analysis of 541 nm luminescence intensity are shown in (b). The statistical data represent mean ± S.D. (*n* = 3). **, *p* < 0.01.

With the development of liver, the albumin synthesis is boosted. Apart from the developmental process, the albumin content seldom changes during homeostasis. However, under acute damage, such as toxic drugs, the liver exhibits reduced albumin synthesis. To further explore the possibility of labeling cells in living animals, we ask whether UCAR nanoprobe could provide quantification and imaging data during liver injury. To model acute liver damage, we established acute liver injury model using carbon tetrachloride (CCl_4_) treatment.^[^ ^25^ ^]^ Treated with CCl_4_, the liver exhibits necrosis and serum albumin reduction, however, the albumin distribution dynamics in liver tissue were largely unknown.^[^ ^26^ ^]^ To address that, we first intraperitoneally injected mice with CCl_4_ or corn oil. After 24 h, UCAR nanoprobe was administrated through splenic injection to increase the liver accumulation.^[^ ^27^ ^]^ Then the mice were sacrificed for lung and liver tissue collection at 4 h post‐injection, followed by sectioning and confocal imaging (**Figure** **5**a).

Before imaging, we collected the serum to quantify the serum albumin with UCAR. Consistent with previous reports, CCl_4_ indeed decreased the serum albumin content (Figure S18, Supporting Information). Then we explored the albumin dynamics at tissue level using UCAR imaging. Excited by 980 nm laser, the lung and liver tissues showed distinct 541 nm luminescence intensity: under both two conditions, the liver, more precisely speaking, hepatocytes displayed much intense 541 nm luminescence than the cells in lung (Figure 5b), which do not express albumin. Meanwhile, for the liver tissue exposed to corn oil and CCl_4_ treatments, the albumin distribution was also different: in control group, the 541 nm luminescence was evenly distributed in liver zones, from pericentral region to periportal region. However, in CCl_4_ group, the 541 nm luminescence dramatically reduced near the pericentral region (zone 3), where numerous small nuclear immune cells penetrated (Figure 5b). The tissue behavior also phenocopied previous reports that CCl_4_ led to necrosis and hepatocyte disfunction in pericentral region.^[^ ^25b^ ^]^ Apart from the dynamic changes at tissue level, the UCAR also gave the robust signal at subcellular level: in enlarged image of corn oil or CCl_4_‐treated liver, the cytoplasm, but not nuclear, showed 541 nm luminescence. The statistical analysis of luminescence intensity quantification further supported those conclusions (Figure 5c). Above all, UCAR nanoprobe could faithfully reveal the dynamic changes of albumin in tissue and subcellular level in situ.

## 3 Discussion

Here, we have developed NIR‐responded UCAR nanoprobe to quantify albumin protein and label albumin in living cells, organoids, and animals. For the proof of principle, we rationally designed core–shell UCNPs connected with antibody‐RBHA to detect albumin by a “turn‐on” strategy. The success of UCAR implies that conjugating UCNPs with specific antibody would be an effective and simple strategy to quantify and image target protein with only one single probe.

Although antibody‐functionalized nanoprobes have been reported,^[^ ^11^ ^]^ the design of FRET‐based antibody sensor was still lacking. Wang et al. utilized thrombin‐aptamer affinity to break down noncovalent interaction between UCNPs and the acceptor,^[^ ^10b^ ^]^ however, the noncovalent nanoparticle was not quite stable for more harsh environment. The UCAR nanoprobe, which sequentially conjugated antibody and RBHA through covalent linkage, displayed stable performance in different biological samples (Figure 2). The key mechanism of UCAR is the binding of antibody–antigen would change the distance between RBHA and UCNPs (Figure 1b), however, unlike noncovalent interactions, the antigen binding unlikely substitutes the conjugated RBHA, the only possibility lies in the conformational change of antibody, which indirectly “pushes” RBHA away from the UCNPs. The antigen binds to the complementarity determining regions on the Fab fragments of antibody, leading to the angular and positional change of first two domains of heavy and light chains.^[^ ^28^ ^]^ As the RBHA is mainly conjugated with N‐terminal of the antibody (except for the lysine residues), we can speculate that conformational change upon antibody–antigen binding would lengthen the distance between RBHA and UCNPs, leading to the restoration of 541 nm luminescence.

The emission spectrum of energy donor UCNPs contained two signal peaks: 541 and 655 nm. Since the absorption spectrum of quencher RBHA only covers the 541 nm, the 655 nm luminescence would serve as reference signals. Although the 541 nm intensity fit in good linear relationship with albumin concentrations, the luminescence ratio of *I*_541_/*I*_655_ could gave constant linear regression equations regardless of magnitude of intensity value. It is also worth noting that the linear regression equations varied in different solutions. In PBS and cell culture medium, the equations were nearly the same, while in the organoid culture medium, the equations, especially the slope were much lower (Figure 2d,g,j). Organoid culture medium is supplied with various growth factors, such as EGF, FGF, HGF, and R‐Spondin, which could bind to the albumin.^[^ ^13^, ^14^ ^]^ Thus, the insufficient exposure of the albumin epitope leads to the insufficient UCAR‐albumin binding, which further reduces the 541 nm luminescence restoration. Therefore, the LIR method could reflect the sensitivity of UCAR in different biological samples.

Liver organoid differentiation system mimics cholangiocyte‐to‐hepatocyte differentiation ex vivo. The golden standard to evaluate hepatocyte differentiation is the albumin expression level. However, traditional methods for albumin quantification include RT‐qPCR (mRNA level), immunofluorescence, and ELISA (protein level), which require remarkable workload. We demonstrated that UCAR could quantify secreted albumin during organoid differentiation in real time, and the detection accuracy is comparable with ELISA (Figure 3e). Therefore, it would be promising that other important secreted factors, such as insulin, thyroxine, and enzymes, could be quantified by our nanoprobe with much convenience. Moreover, by quantification of the secreted factors in real time, UCAR would record the fast‐changing cell status in response to the stimulus, such as pH, temperature, and drugs, facilitating the cell engineering researches and clinical diagnostics.

In contrast with the in vitro quantification, which requires homogenous distribution of target protein, the imaging emphasizes on heterogenous distribution of target protein at organ and cellular level. By combining liver injury model and living animal administration of UCAR nanoprobe through splenic injection, we observed the dynamics of albumin in liver through in situ imaging of albumin (Figure 5). After CCl_4_ treatment, the overall intensity of albumin in liver was reduced, especially in the pericentral region. It is worth noting that scattered hepatocytes surrounding central vein still showed robust 541 nm luminescence. One explanation is that the CCl_4_ first damages the zone 2 region, not zone 3 of the liver lobule. Since regeneration of cytokines‐TNF*α* rapidly increased upon CCl_4_ treatment/PHx in 6 h,^[^ ^25^, ^29^ ^]^ we can also speculate those albumin positive cells in zone 3 were newly regenerated hepatocytes. To improve the feasibility of living animal application, we performed hydrodynamic tail vein injection to deliver the nanoprobe. The results showed that compared with mock group (saline injected), the UCAR injected group exhibits robust signal in hepatocyte cytoplasm (Figure S19, Supporting Information), which was consistent with splenic injection data. Although the living animal labeling method still needs tissue fixation and sectioning, we believe that with the advance of in vivo imaging facilities, the UCAR nanoprobe would allow the in vivo imaging of endogenous protein at cellular level in near future. Even so, UCAR imaging has significant advantages over traditional immunofluorescence method, which usually needs two antibodies and laborious staining/wash protocols. Besides, since we only used one fluorescent channel, there would be enough remaining channels for simultaneous imaging of multiple proteins by engineering the UCNPs and antibody. Furthermore, together with morphology‐correlated organelle identification, more precise subcellular protein location would be achieved.

In summary, we have designed a dual‐functional nanoprobe for protein quantification and imaging. This nanoprobe is characterized by UCNPs‐antibody‐RBHA, which is short for UCAR. The luminescence intensity of UCNPs is greatly quenched by RBHA with a high quenching rate of 98% based on FRET strategy. Exampled by albumin antibody, the UCAR achieves a detection limit as low as 0.053 ng mL^−1^ and shows an excellent linear response to albumin in a broad range (5–315 ng mL^−1^) in different media. Furthermore, we demonstrate that UCAR nanoprobe also can be used for bio‐imaging and albumin detection in living cell and liver organoids, as well as mouse liver, showing great potential in protein tracking in vivo and clinical diagnosis.

## 4 Experimental Section

### Chemicals and Reagents

1‐(3‐Dimethylaminopropyl)‐3‐ethylcarbodiimide hydrochloride (EDC, 98%), *N*‐hydroxysuccinimide (NHS, 98%), ethanol (99.5%), rose‐bengal (RB, 90%), and ammonium fluoride (NH_4_F, 98%) were purchased from Beijing Chemical Regent Co., Ltd.; PBS (pH = 7.2–7.4) was purchased from Beijing Solarbio Co., Ltd.; YCl_3_·6H_2_O (99.99%), YbCl_3_·6H_2_O (99.99%), ErCl_3_·6H_2_O (99.99%) were purchased from Beijing HWRK Chemical Co., Ltd.; methanol (≥99.5%), 6‐hexanoic acid (HA, 97%), NaOH (≥96%), *N*,*N*‐dimethylformamide (DMF, 99.9%), oleic acid (OA, 90%), polyvinylpyrrolidone (PVP), and 1‐octadecence (ODE, 90%) were purchased from Aladdin; nitrosonium tetrafluoroborate (NOBF_4_, 95%), polyethylenimine (PEI, 25 kDa), 4,6‐diamidino‐2‐phenylindole dihydrochloride (DAPI, ≥95.0%) were purchased from Thermo Fisher; Dulbecco's modified Eagle's medium (DMEM) and fetal bovine serum (FBS) were purchased from Bestbio Co. (Shanghai, China); the albumin antibody (production code # BAS7673‐0) was purchased from Shanghai Yuduo Biotechnology Co., Ltd.; organoid culture reagents, including advanced DMEM/F12, B27, N2, penicillin/streptomycin, *N*‐acetylcysteine, and GlutaMax were purchased from bioGenous and Thermo Fisher; growth factors including EGF, FGF10, HGF, R‐Spondin1, TNF*α*, and Gastrin I were purchased from OrganRegen. Small molecules including CHIR99021, A83‐01, DAPT, and dexamethasone were purchased from MCE.

### Cell Lines, Organoids, and Mice

HaCat cells (human keratinocytes cell line) and HepG2 cells (human hepatocellular carcinomas cell line) were obtained from the Institute of Biochemistry and Cell Biology, Chinese Academy of Sciences. Mouse liver ductal and hepatocyte organoid were isolated and cultured as previously reported.^[^ ^21^, ^30^ ^]^ The C57BL/6 mice were purchased from Shanghai Research Center for Model Organism. All animal studies were performed in accordance with the relevant guidelines and under the approval of the Institutional Animal Care and Use Committee of Fudan University (Ethical approval number: 2020JS038).

### Preparation of NaYF_4_:Yb^3+^/Er^3+^ and NaYF_4_:Yb^3+^/Er^3+^@NaYF_4_ Nanoparticles

The solvothermal method was used to prepare NaYF_4_:Yb^3+^/Er^3+^. YCl_3_ (1 m, 0.78 mL), YbCl_3_ (1 m, 0.2 mL), ErCl_3_ (0.1 m, 0.02 mL) were added into a flask with three necks. The mixed solution was heated to 90 °C until it was evaporated completely. After that, OA (6 mL) and ODE (15 mL) were added into the flask and the solution was heated to 130 °C until the light yellow and translucent solution was formed. Then the solution was cooled to room temperature and the methanol solution containing sodium hydroxide (0.1 g) and ammonium fluoride (0.14816 g) was added into it. The mixed solution was heated to 70 °C and hold for 30 min and in vacuum for 10 min. Then the solution was further heated to 300 °C and hold for 1 h under nitrogen airflow environment. Once again, the solution was cooled to room temperature, the excess ethanol was added to precipitate the nanoparticles (NaYF_4_:Yb^3+^/Er^3+^). The nanoparticles were dispersed in 22.5 mL cyclohexane after washing and centrifugation. The preparation process of the core–shell structure (NaYF_4_:Yb^3+^/Er^3+^@NaYF_4_) was similar to that of the core, except that the sensitizer (Yb^3+^) and activator (Er^3+^) were not added.

### Nanoparticle Characterization

The transmission electron microscope (TEM) images were performed utilizing an FEI Tecnai G2 S‐Twin with a field‐emission gun operating at 200 kV. The crystal structure was determined by X‐ray powder diffraction (Bruker) with Cu Ka radiation (*λ* = 0.15405 nm). FTIR spectra were performed on a Vertex Perkin‐Elmer 580BIR spectrophotometer (Bruker) with the KBr pellet technique. TG was performed by using a Netzsch STA 409 thermoanalyzer with a heating rate of 10 °C min^−1^ in nitrogen. The emission spectra were detected by a fluorescence spectrophotometer (Hitachi, F‐7000). And the absorption spectra were determined by an UV spectrophotometer (UV2600, Shimadzu).

### Step‐Wised Synthesis of UCAR

For the preparation of UCNPs‐PEI, the DMF (5 mL) and cyclohexane (3 mL) were added into DMF solution containing NOBF_4_ (100 mg) and stirred for 10 min. The cyclohexane solution (7.5 mL) containing UCNPs was further added into the solution and the solution was stirred for 30 min. Then the UCNPs‐BF_4_ nanoparticles were obtained by centrifugation and redispersed in DMF solution (5 mL). After that, the DMF solution (5 mL) containing PEI (100 mg) was added into the UCNPs‐BF_4_ solution and the solution was stirred for 12 h to form the UCNPs‐PEI. The UCNPs‐PEI was obtained by washing in DMF and deionized water in sequence and centrifugation (12 000 *g* × 10 min). Finally, the UCNPs‐PEI was dispersed in PBS (22.5 mL).

For preparation of UCNPs‐Ab, the EDC (2 mg mL^−1^, 200 µL), NHS (2 mg mL^−1^, 100 µL), PVP (4 mg mL^−1^, 100 µL), and Ab were centrifuged and shook for 2 h. The UCNPs‐PEI (200 µL) was added into the solution and incubated overnight to form the UCNPs‐Ab.

For the preparation of UCNPs‐Ab‐RBHA, the RB (100 mg) and HA (20 mg) were added into a mixed solution with *V*_acetone_:*V*_water_ = 7:3 and stirred for 24 h at 75 °C. The rotary evaporator was used to volatilize acetone, and the RBHA was extracted from the mixture of water and ethyl acetate. Then the dry RBHA was obtained by lyophilization. The EDC (2 mg mL^−1^, 200 µL), NHS (2 mg mL^−1^, 100 µL), and RBHA (250 µg mL^−1^, 200 µL) were centrifuged and shook for 2 h to form the mixed RBHA solution.

The BSA was added into PBS solution containing UCNPs‐Ab and incubated for 30 min. After the solution was ultrasound for 1 min, the mixed RBHA solution was added into the solution and the solution was incubated for 2 h to obtain UCAR.

### The Calculation of the Amount of RBHA, Encapsulation, and Drug Loading of RBHA on the Surface of UCNPs‐Ab

The obtained UCNPs‐Ab‐RBHA was dispersed in deionized water (0.1 mL). A UV‐2600 Shimadzu spectroscope was used to detect the UV‐visible‐near infrared spectrum of the sample. The concentration of RBHA could be calculated according to the fitting equation of *y* = 0.023*x* + 0.153 in Figure S8b in the Supporting Information.

The calculated concentration of RBHA was 477.4 µg mL^−1^, and the calculated amount of RBHA was 47.74 µg.

The encapsulation and drug loading rate could be calculated by the following formula^[^ ^22^ ^]^

(1) $$\eta_{1}=\frac{m_{1}}{m_{2}}\times \;100\%$$

(2) $$\eta_{2}=\frac{m_{1}}{m_{2}+m_{3}}\times \;100\%$$

here the *η*_1_ and *η*_2_ denote the encapsulation and drug loading rate, respectively. The *m*_1_ (47.74 µg) is the loading amount of RBHA, *m*_2_ denotes the total amount of RBHA (50 µg), and *m*_3_ represents the amount of UCNPs‐Ab (1.06 mg).

### The Calculation of the UCAR's Detection Limit to Albumin

The detection limit of UCAR could be calculated as follows

(3) $$x=\frac{3\sigma -b}{k}$$

here the *x* represents the detection limit, the *k* and *b* denote the slope and intercept of fitting line, respectively. And *σ* is the standard deviation of control group.

### Liver Ductal Organoid Differentiation

For mouse liver ductal organoid differentiation, organoids cultured in EM were refreshed with DM, which contained 50 ng mL^−1^ EGF, 100 ng mL^−1^ FGF10, 10 × 10^−9^ m gastrin, 50 × 10^−9^ m A83‐01, and 10 × 10^−6^ m DAPT. The organoids were supplied with fresh DM each day for up to 9 days. After that, the differentiation medium was supplemented with dexamethasone (3 × 10^−6^ m), in which the organoids were cultured for additional 3 days.

### qRT‐PCR and ELISA

Total RNA was extracted with RNeasy Mini Kit (Qiagen) according to the manufacturer's instruction. cDNA was synthesized with GoScript Reverse Transcription System (Promega). qRT‐PCR reactions were performed with GoTaq qPCR Master Mix (Promega) in triplicates on CFX96 Touch System (BioRad). Primer sequences are listed in Table S2 in the Supporting Information. Albumin ELISA was performed according to the manufacturer's instructions (Abcam, ab108792; Bethyl, E88129).

### UCAR‐Mediated Albumin In Situ Imaging in Cells and Organoids

The HepG2/HaCat cells were seeded in 24‐well plate inserted with round coverslip with the cell density of 10^5^ cells per well. Then the cells were incubated with UCNPs‐Ab‐RBHA (10 × 10^−6^ m) for 12 h. Subsequently, the 4% formaldehyde was used to fix the cells and the DAPI was used to dye nucleus. Imaging was performed by two‐photon microscopy (Leica TCS SP8 DIVE FALCON).

The liver ductal and hepatocyte organoids were cultured in 3D Matrigel until the diameter of organoid reached 50–100 µm. The process of nucleus dye in organoids and imaging of UCNPs‐Ab‐RBHA in organoids were performed as in cells as mentioned above.

### UCAR‐Mediated Albumin In Situ Imaging in Mouse Tissues

For establishing mouse liver injury model, C57BL/6 mice (6–8 weeks, male) were intraperitoneally injected with carbon tetrachloride (CCl_4_, diluted with corn oil) at the dosage of 1 mL kg^−1^ body weight. Control mice were received equal volume of corn oil.

After 24 h of CCl_4_ administration, both treated and control mice were subjected to splenic injection of UCAR nanoparticles through surgical operations. Each mouse was injected with 200 uL UCAR (0.6 × 10^−3^ m). After 4 h, mice were sacrificed to collect the lung and liver tissue followed by overnight fixation in 4% paraformaldehyde. Then the fixed tissues were embedded with optimal cutting temperature compound followed by frozen sectioning. Before imaging, the tissue sections were incubated with the DAPI to dye the nucleus. Imaging was performed by two‐photon microscopy (Leica TCS SP8 DIVE FALCON). The luminescence intensity was quantified by Image J software.

All animal studies were performed in accordance with the relevant guidelines and under the approval of the Institutional Animal Care and Use Committee of Fudan University (Ethical approval number: 2020JS038).

### Statistical Analysis

All values were represented as mean ± S.D. Student's *t* test and two‐way analysis of variance were used to compare differences between two groups as indicated. Statistical analysis was performed with the GraphPad Prismsoftware.

## Conflict of Interest

The authors declare no conflict of interest.

## Author Contributions

G.L. and J.W. contributed equally to this work. G.L., J.W., J.W., S.X., and B.Z. conceived the study; G.L., J.W., X.L., M.T., Z.W., C.S., W.S., C.L., X.L., S.T., and E.L. performed the experiments; J.W., S.X., and B.Z. supervised the work; G.L., J.W., J.W., S.X., and B.Z. wrote the manuscript.

## Supporting information

Supporting Information

## Data Availability

The data that support the findings of this study are available from the corresponding author upon reasonable request.
