# Supplementary material for: Near‐Infrared‐Responded High Sensitivity Nanoprobe for Steady and Visualized Detection of Albumin in Hepatic Organoids and Mouse Liver
Source: Adv Sci (Weinh). 2022 Jul 19;9(26):2202505. doi: 10.1002/advs.202202505 (PMC9475548; doi:10.1002/advs.202202505)
Supplement: Supplementary file 1 — Supporting Information [file ADVS-9-2202505-s001.pdf]

ADVANCED SCIENCE Open Access

Supporting Information

for *Adv. Sci.*, DOI 10.1002/advs.202202505

Near-Infrared-Responded High Sensitivity Nanoprobe for Steady and Visualized Detection of Albumin in Hepatic Organoids and Mouse Liver

Guofeng Liu, Jinsong Wei, Xiaoyu Li, Meng Tian, Zhenxing Wang, Congcong Shen, Wan Sun, Chonghui Li, Xuewen Li, Enguang Lv, Shizheng Tian, Jihua Wang, Shicai Xu* and Bing Zhao**

Supporting Information

Near-infrared-responded high sensitivity nanoprobe for steady and visualized detection of albumin in hepatic organoids and mouse liver

Guofeng Liu et al

Figure S1-S19

Table S1-S2

Figure S1

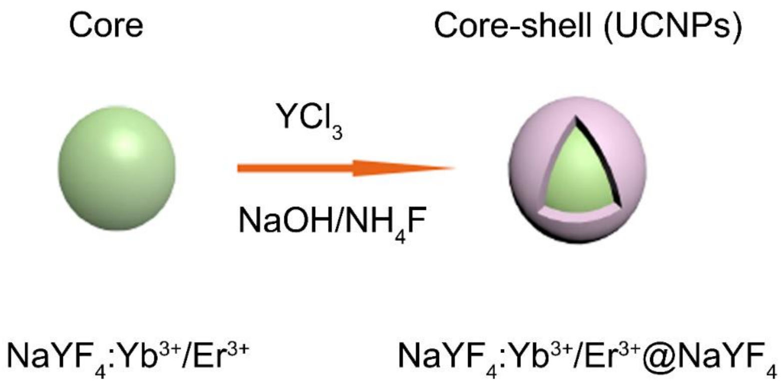

Figure S1. **Scheme of core-shell structured UCNPs synthesis**.

Figure S2

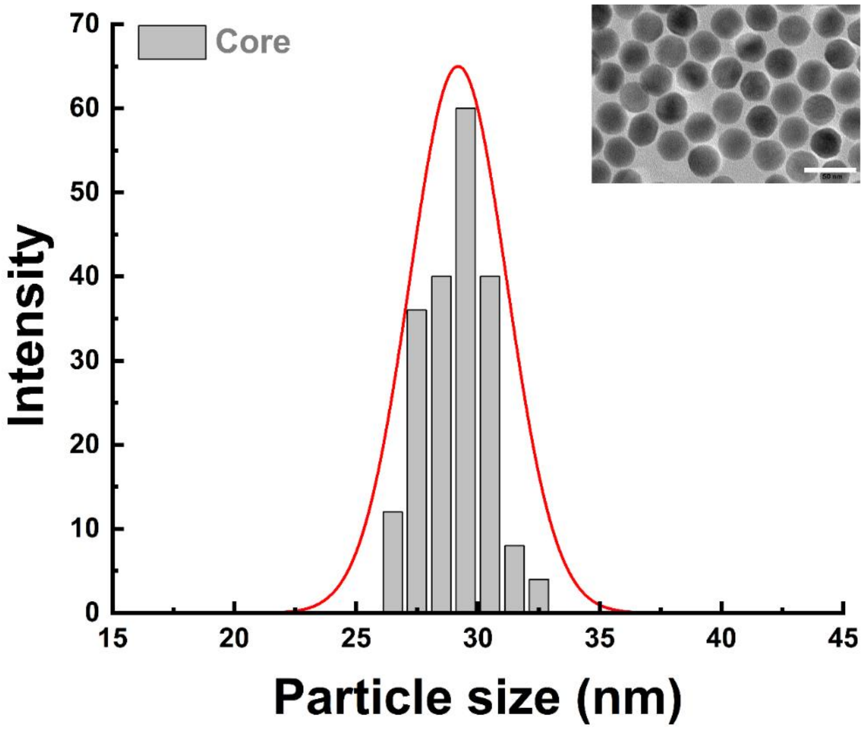

Figure S2. The size distribution and TEM image of Core nanoparticles ($NaYF_4$: Yb^{3+}/Er^{3+}), and the average particle size was about 29 nm. Scale bar=50 nm.

Figure S3

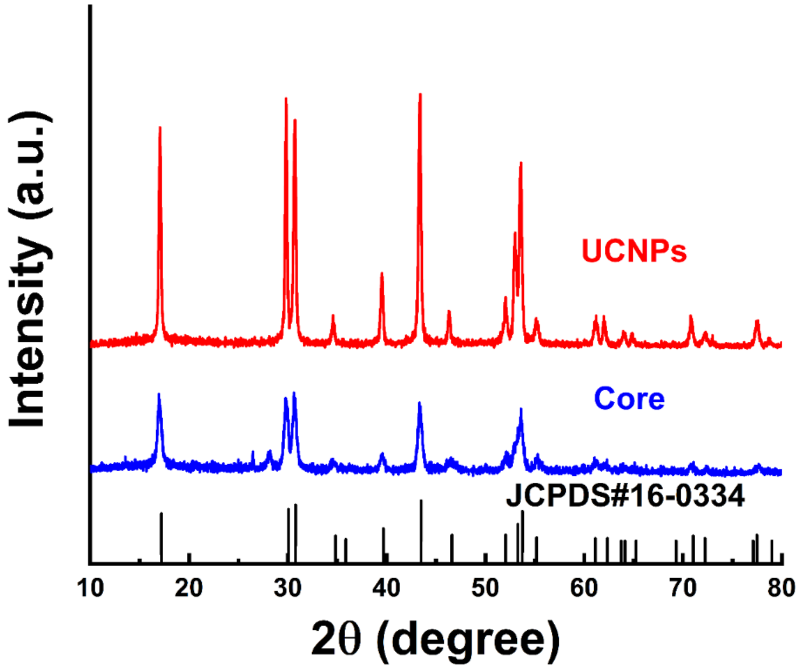

Figure S3. The XRD patterns of core and UCNPs (NaYF4: Yb3+/Er3+@NaYF4). The XRD phases of core and UCNPs were in good agreement with the standard card, which proved that the synthesized core and UCNPs nanoparticles were pure hexagonal phase.

Figure S4

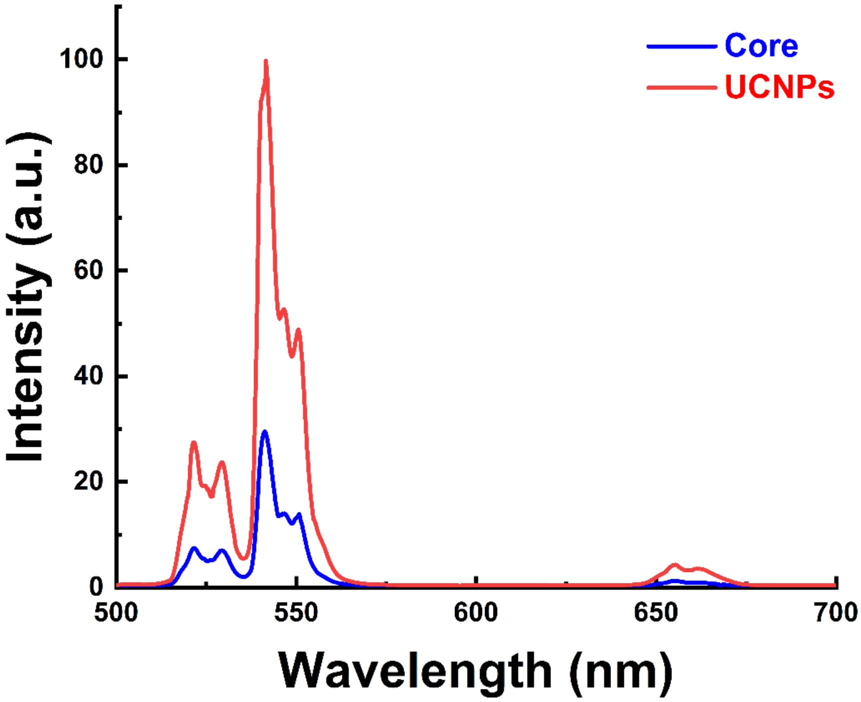

Figure S4. The emission spectra of core and UCNPs under 980 nm laser irradiation (1 W/cm2). The fluorescence intensity of UCNPs with inert shell was greatly enhanced due to the reduction of surface quenching.

Figure S5

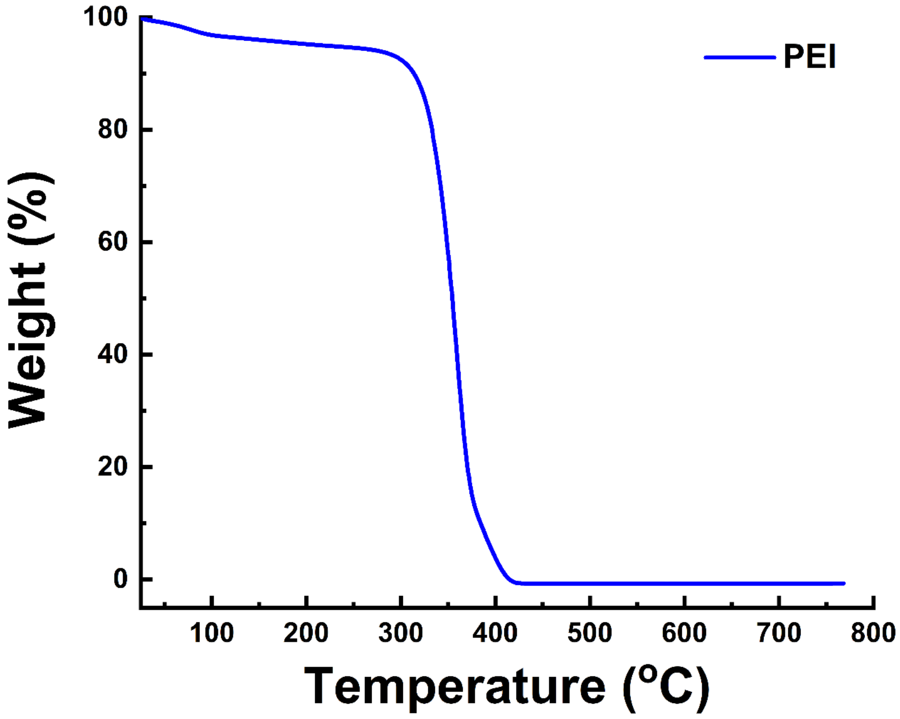

Figure S5. The TG of PEI from 20 to 800°C at air.

Figure S6

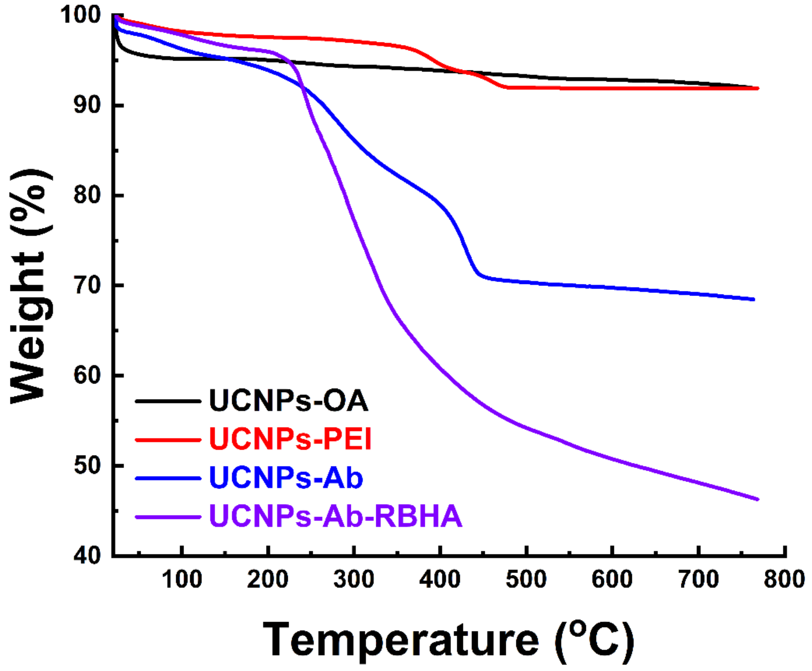

Figure S6. The TG of UCNPs-OA, UCNPs-PEI, and UCNPs-Ab from 20 to 800℃ at air. The curves indicated weight loss of the conjugated organic groups during each step of UCAR synthesis. The slight weight decreases of UCNPs-OA (black) and UCNPs-PEI (red) indicated the oleic acid (OA) and PEI. The dramatic dive of UCNPs-Ab (blue, 32.5% loss) and UCNPs-Ab-RBHA (purple, 54.5% loss) at 300-500℃ suggested that antibody, BSA and RBHA were also carried on the surface of nanoparticles.

Figure S7

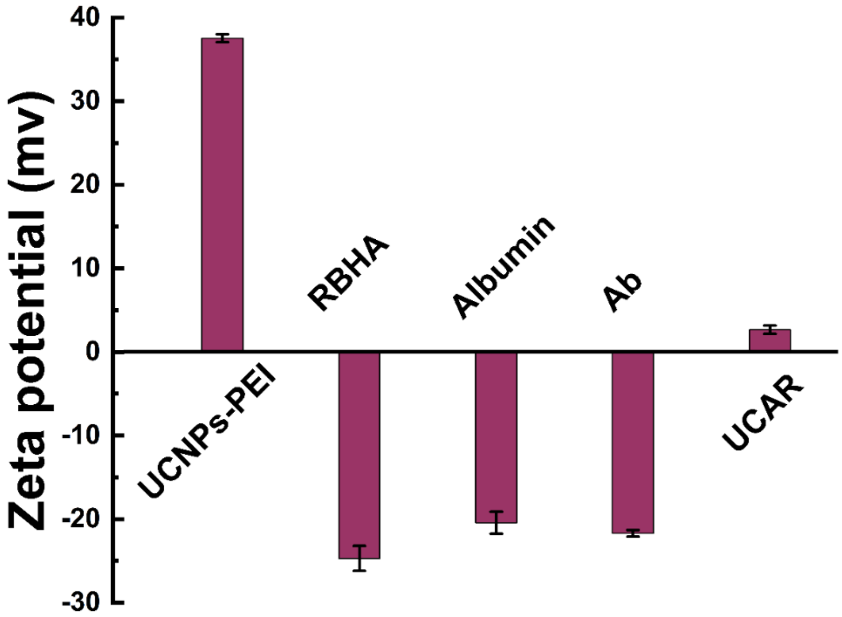

Figure S7. The zeta potential of UCNPs-PEI, RBHA, Albumin, Ab and UCAR. Data were represented as mean ± S.D. (n=3).

Figure S8

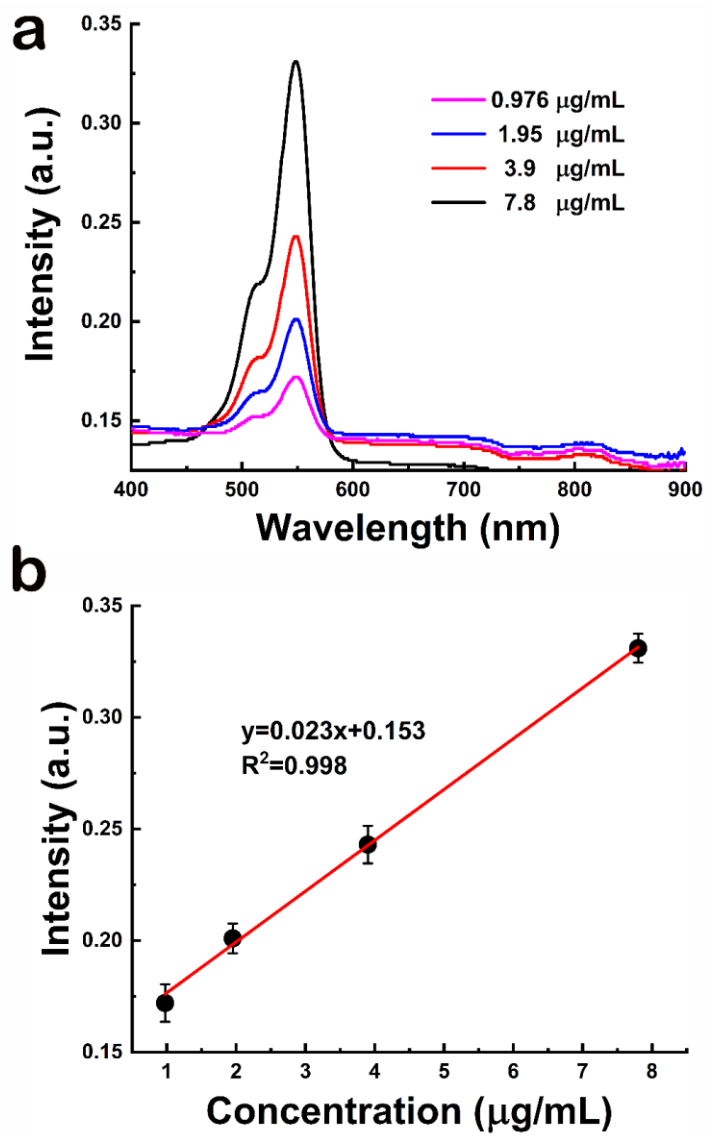

Figure S8. The linear relationship between RBHA absorption peak intensity and concentrations. (a) The absorption spectra of RBHA under different concentrations; (b) The absorption peak intensity of RBHA changed linearly with the increase of concentration. Therefore, the encapsulation efficiency and drug loading rate of RBHA on the surface of UCNPs could be calculated according to the linear regression. Data were represented as mean ± S.D. (n=3)

Figure S9

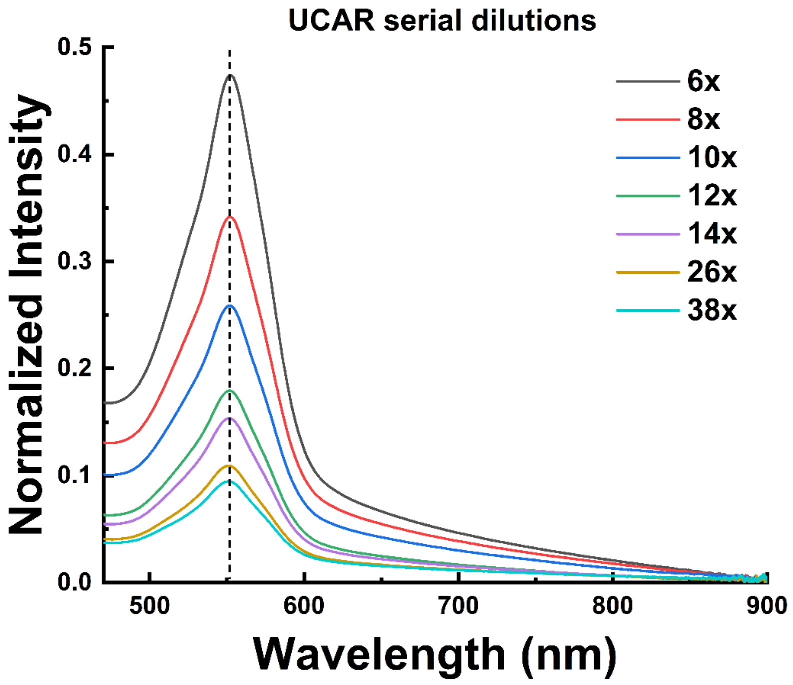

Figure S9. The absorption curves of UCAR serial dilutions in PBS solution. For the calculation of RBHA encapsulation efficiency, the UCAR nanoparticles were diluted in PBS solution as indicated fold (6x, 8x, 10x 12x, 14x, 26x and 38x), and the absorption spectra were recorded by an UV spectrophotometer (UV2600, Shimadzu).

Figure S10

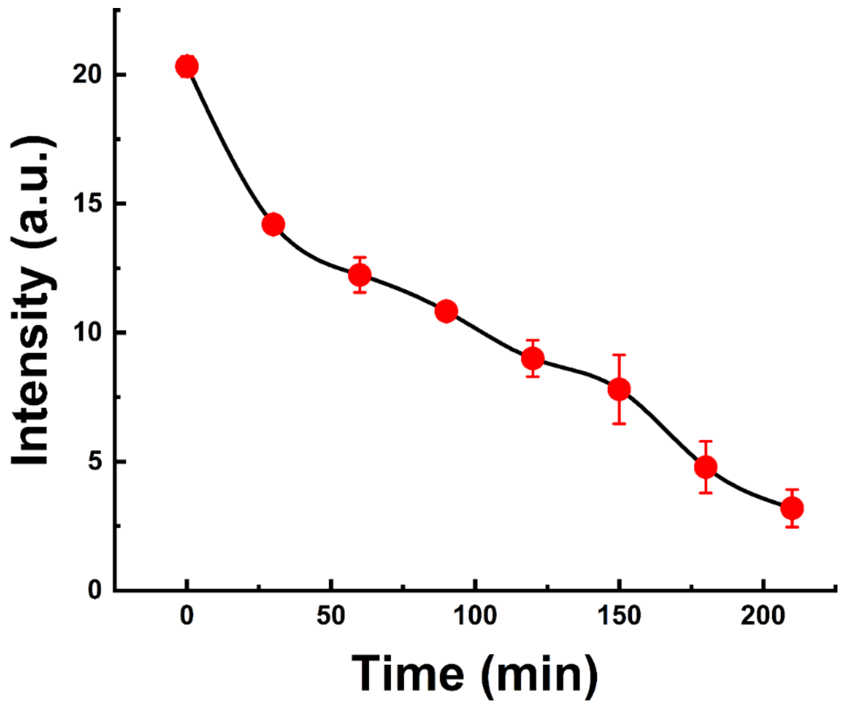

Figure S10. The 541nm luminescence intensity of UCNPs-Ab after incubated with RBHA for indicated times. As the incubation time prolonged, the more RBHA would bind to UCNPS-Ab, leading to the decrease of its luminescence intensity. Data were represented as mean ± S.D. (n=3).

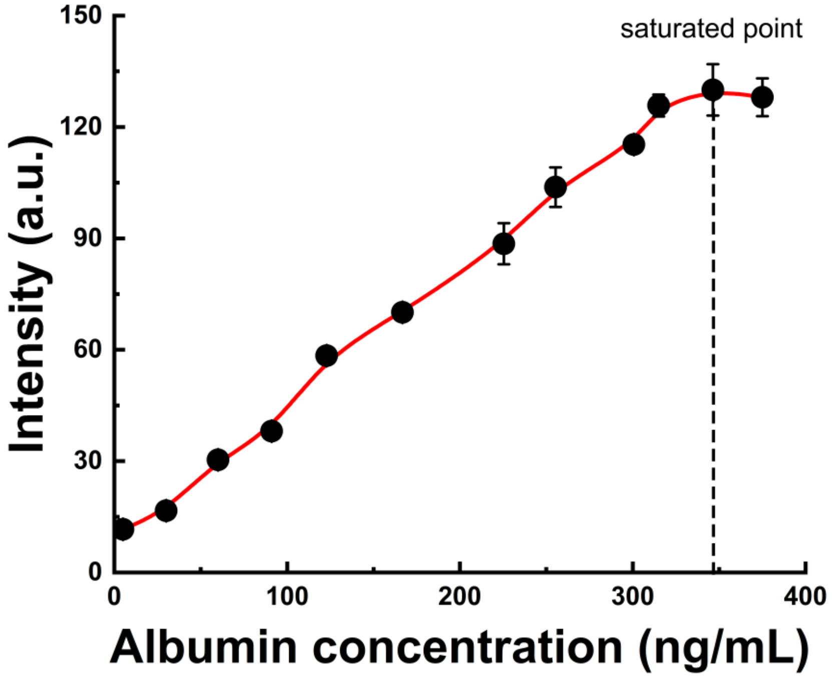

Figure S11. Quantification of max albumin binding content onto the UCAR nanoprobe. To determine the maximum albumin binding content, the UCAR (mM) was incubated with albumin of increasing concentrations (5-400 ng/mL) at room temperature for 2hours, then the 541 nm luminescence was measured. Data were represented as mean ± S.D. (n=3).

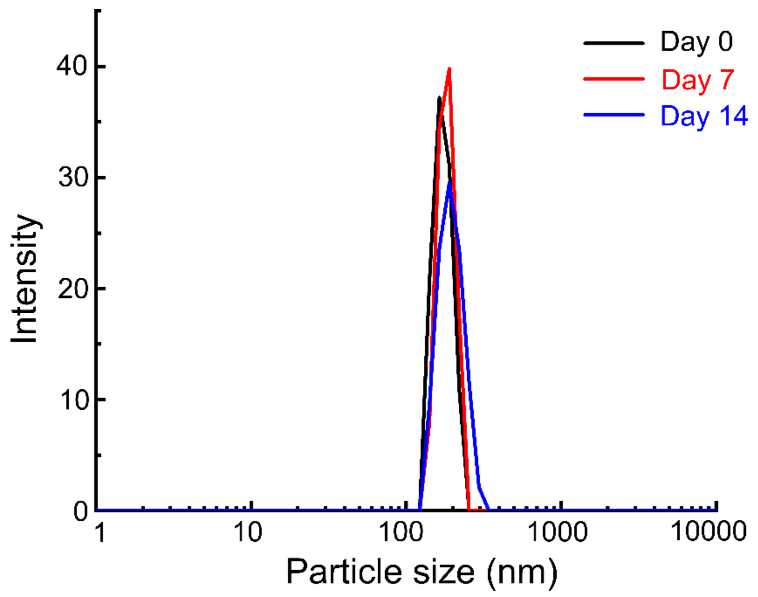

Figure S12. Hydrodynamic diameter of UCAR in serum at different time points.

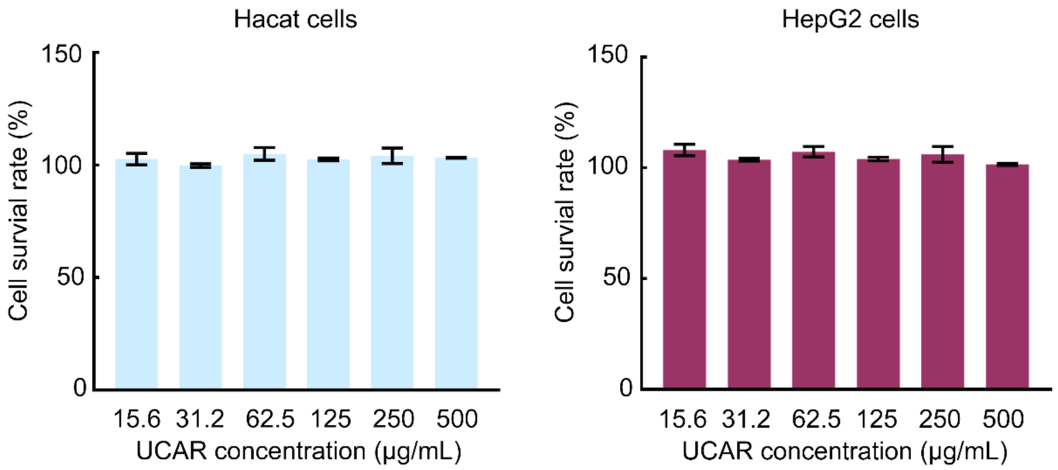

Figure S13. UCAR exhibited extremely low cytotoxicity in cells. Hacat and HepG2 cells were incubated with UCAR at indicated concentrations for 24 hours, then subjected to CCK-8 assay to determine the cell survival rates. Data were represent as mean ± S.D. (n=3).

Figure S11

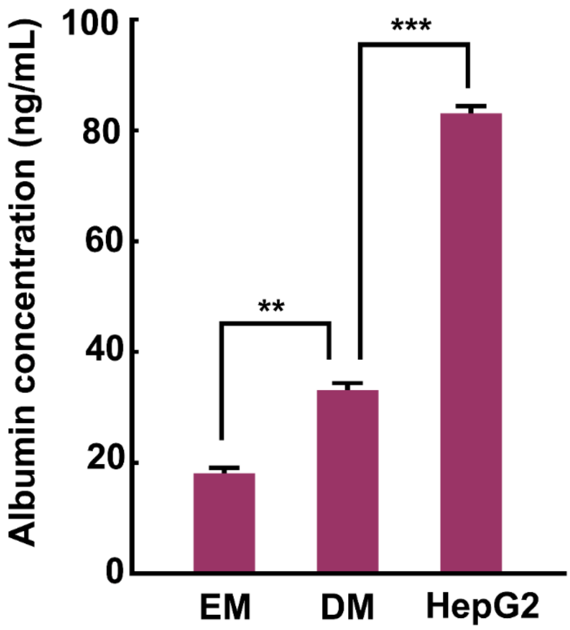

Figure S14. ELISA quantification of albumin concentration secreted by liver organoid in EM and DM, as well as HepG2 cells. The statistical data represent mean±S.D. (n=3). **, p<0.01. ***, p<0.001.

Figure S12

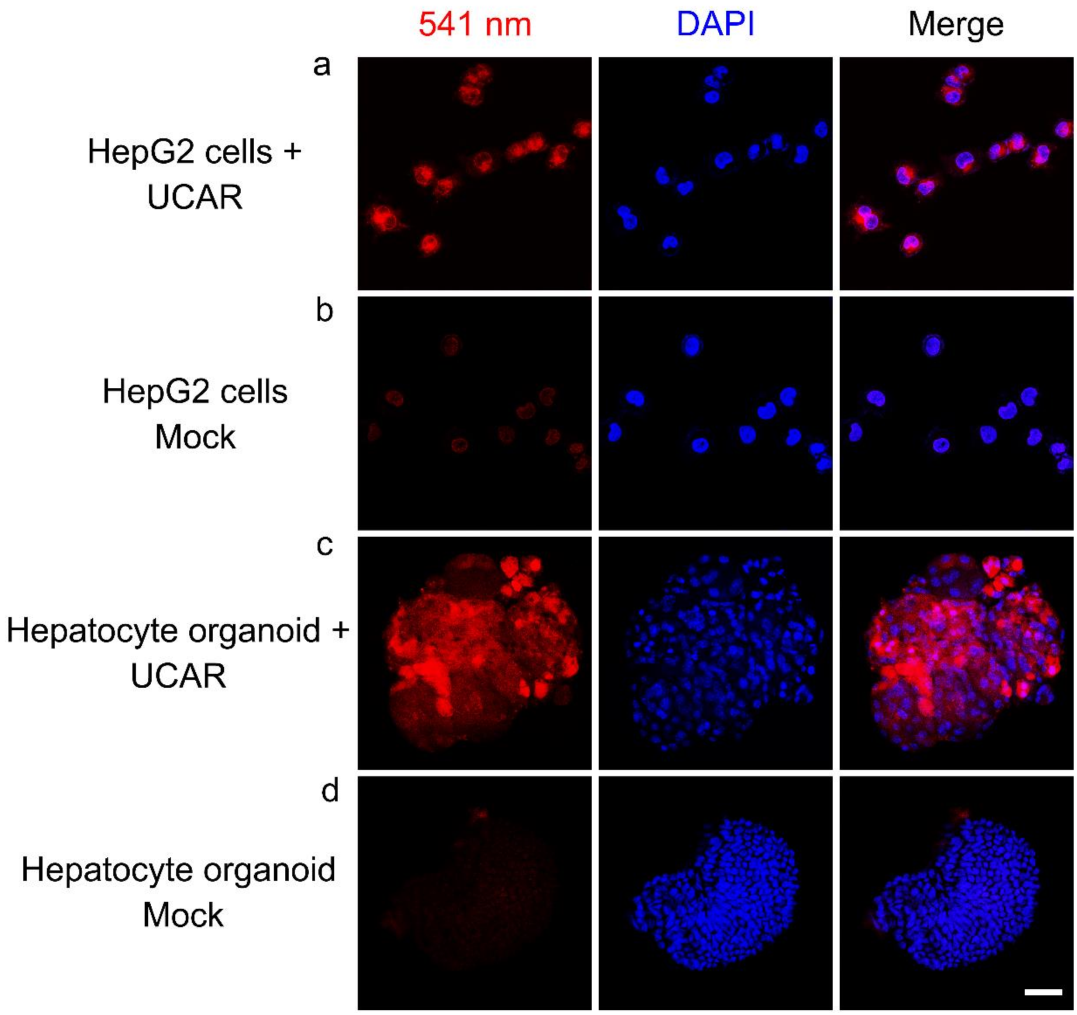

Figure S15. The bio-imaging in HepG2 cells and hepatocyte organoids with or without UCAR. The HepG2 cells with UCAR (a), HepG2 cells without UCAR (b), hepatocyte organoid with UCAR (c), and hepatocyte organoid without UCA (d) were incubated with UCAR for 3 hours. The released 541 nm fluorescence was shown by red color, and 405 nm DAPI fluorescence was shown by blue color. Scale bar= 50 µm.

Figure S13

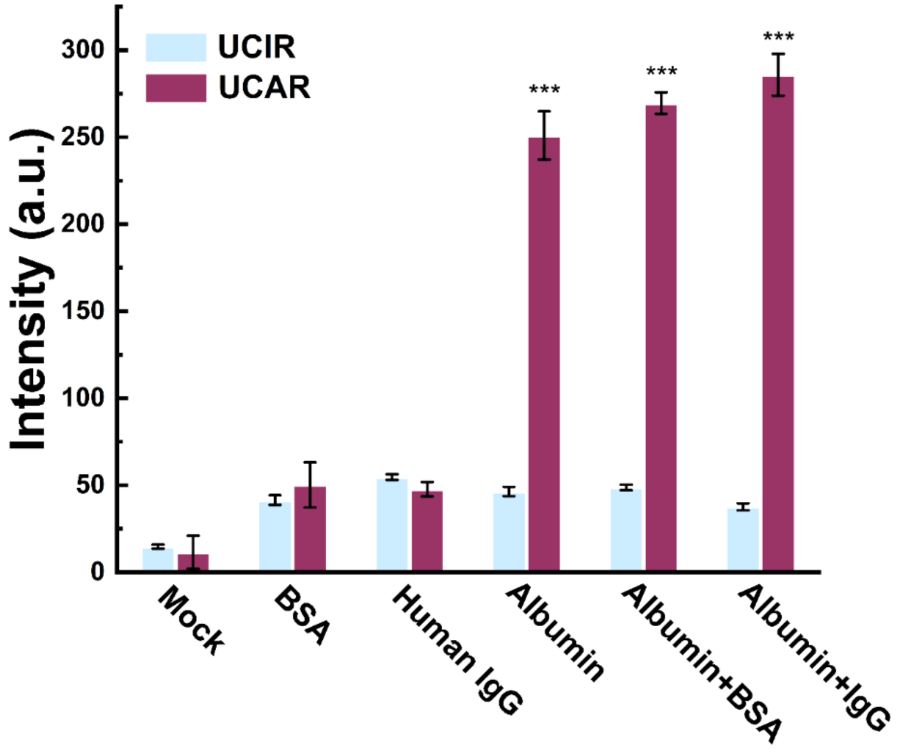

Figure S16. The specificity of nanoprobe to albumin. To detect the specificity of nanoprobe, we synthesized the UCNPs-IgG-RBHA (UCIR) as the control nanoprobe. Then, the UCIR or UCAR was incubated with bovine serum albumin (BSA), human IgG, albumin, albumin+BSA and Albumin+IgG, respectively. After incubation, the 541 nm luminescence intensity was recorded. The volume and concentration of each substrate was listed as follows: Mock (150 μL PBS), IgG (150 μL, 1 μg/mL), BSA (150 μL, 0.75 μg/mL), albumin (150 μL, 0.75 μg/mL), albumin+BSA(100 μL albumin (1 μg/mL) plus 50 μL BSA (μg/mL)), and albumin+IgG (100 μL albumin (1 μg/mL) plus 50 μL IgG (1 μg/mL)). The UCAR (100 μL) and UCIR (100 μL) were added into detection substances (150 μL) and incubated at room temperature for 2 hours. The statistical data represent mean ± S.D. (n=3). ***, p<0.001.

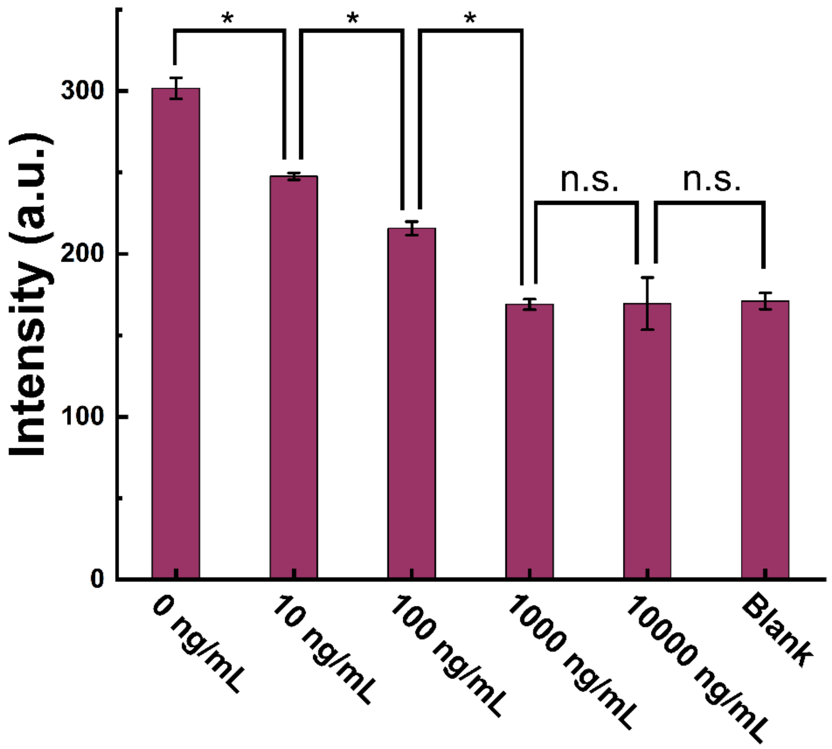

Figure S17. Albumin antibody neutralized the nanoprobe in a dose-dependent manner. The hepatocyte organoid conditional medium was incubated with UCAR and albumin antibody with different concentrations, ranging from 0 ng/mL to 1000 ng/mL, then the luminescence intensity at 541 nm was determined. The statistical data represent mean ± S.D. (n=3). *, p<0.05, n.s., no significant difference.

Figure S14

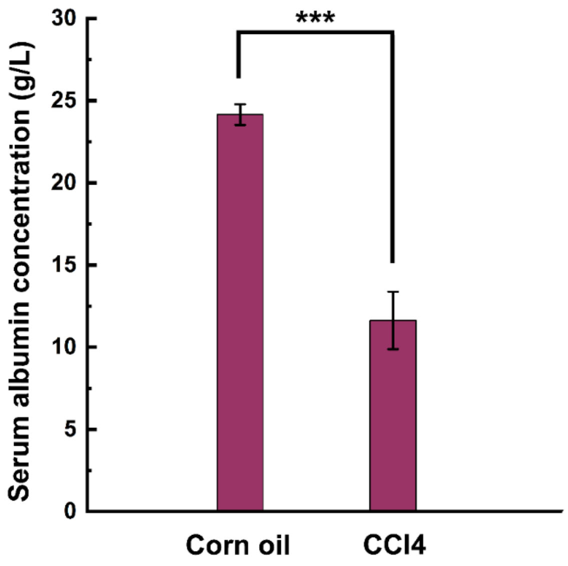

Figure S18. **Quantification of mouse serum albumin with UCAR**. Mice were received one does CCl4 (1 mL/kg, diluted with corn oil) treatment through intraperitoneal injection. After 24 hours, mouse serum was collected for UCAR mediated albumin quantification. The statistical data represent mean ± S.D. (n=3). ***, p<0.001.

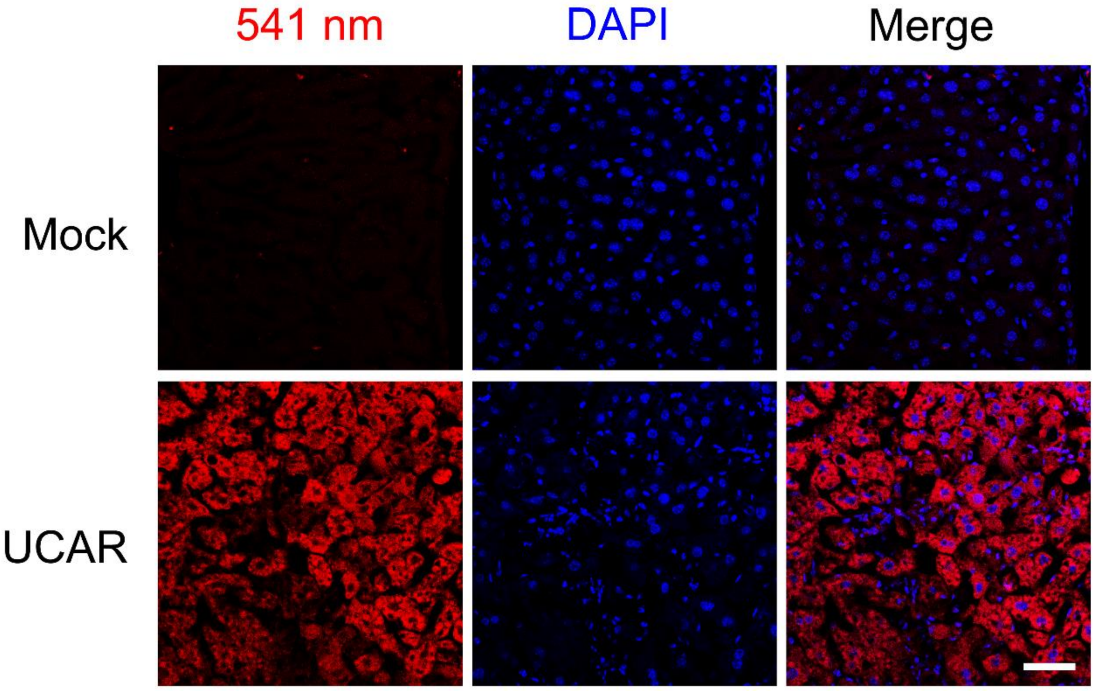

Figure S19. UCAR mediated in situ albumin imaging through hydrodynamic tail vein injection. Mice were injected with saline (mock) or UCAR through tail vein injection. After 4 hours, the liver was perfused and fixed with 4% PFA. Then the liver sections were subjected to 980 nm excitation using two-photon microscope. UCAR released 541 nm luminescence was shown by red color, and 405 nm DAPI was shown by blue color. A representative result of three independent experiments is shown. Scale bar=50 μm.

Table S1. The detection sensitivities (ng/mL) of albumin in different solutions by luminescence intensity ratio (LIR) methods

Solution environment	PBS	Cell culture medium	Organoid culture medium
**LIR (I_{541}/I_{655})**	0.0346	0.0325	0.0066

Table S2. Primers for qRT-PCR

Name	Sequence (5'-3')
**mAlb-F**	ATGTTACCAAGTGCTGTAGT
**mAlb-R**	AATCTGCTTCTCCTTCTCTG
**mTtr-F**	CTCACCACAGATGAGAAG
**mTtr-R**	GGCTGAGTCTCTCAATTC
**mCyp3a11-F**	TACTGTGATGGAGATGGAATAC
**mCyp3a11-R**	GGTGAAGAGCATAAGATGGA
**mMup20-F**	GCGAGGAGCATGGAATCGTT
**mMup20-R**	TGATCCTGGAGTCCTGGTGAGA
**mSox9-F**	CGGAACAGACTCACATCTCTCC
**mSox9-R**	GCTTGCACGTCGGTTTTGG
**mH3-F**	TCCGCGTTTCCGTAGTACAAC
**mH3-R**	GGGGCTGGAGCTGATTTGG
